# Competence and regulatory interactions during regeneration in plants

**DOI:** 10.3389/fpls.2014.00142

**Published:** 2014-04-11

**Authors:** Ajai J. Pulianmackal, Abdul V. K. Kareem, Kavya Durgaprasad, Zankhana B. Trivedi, Kalika Prasad

**Affiliations:** School of Biology, Indian Institute of Science Education and ResearchThiruvananthapuram, India

**Keywords:** competence for regeneration, *De novo* organ initiation, callus, epigenetic modifications, auxin, cytokinin

## Abstract

The ability to regenerate is widely exploited by multitudes of organisms ranging from unicellular bacteria to multicellular plants for their propagation and repair. But the levels of competence for regeneration vary from species to species. While variety of living cells of a plant display regeneration ability, only a few set of cells maintain their stemness in mammals. This highly pliable nature of plant cells in-terms of regeneration can be attributed to their high developmental plasticity. *De novo* organ initiation can be relatively easily achieved in plants by proper hormonal regulations. Elevated levels of plant hormone auxin induces the formation of proliferating mass of pluripotent cells called callus, which predominantly express lateral root meristem markers and hence is having an identity similar to lateral root primordia. Organ formation can be induced from the callus by modulating the ratio of hormones. An alternative for *de novo* organogenesis is by the forced expression of plant specific transcription factors. The mechanisms by which plant cells attain competence for regeneration on hormonal treatment or forced expression remain largely elusive. Recent studies have provided some insight into how the epigenetic modifications in plants affect this competence. In this review we discuss the present understanding of regenerative biology in plants and scrutinize the future prospectives of this topic. While discussing about the regeneration in the sporophyte of angiosperms which is well studied, here we outline the regenerative biology of the gametophytic phase and discuss about various strategies of regeneration that have evolved in the domain of life so that a common consensus on the entire process of regeneration can be made.

## Introduction

**Figure 1:**
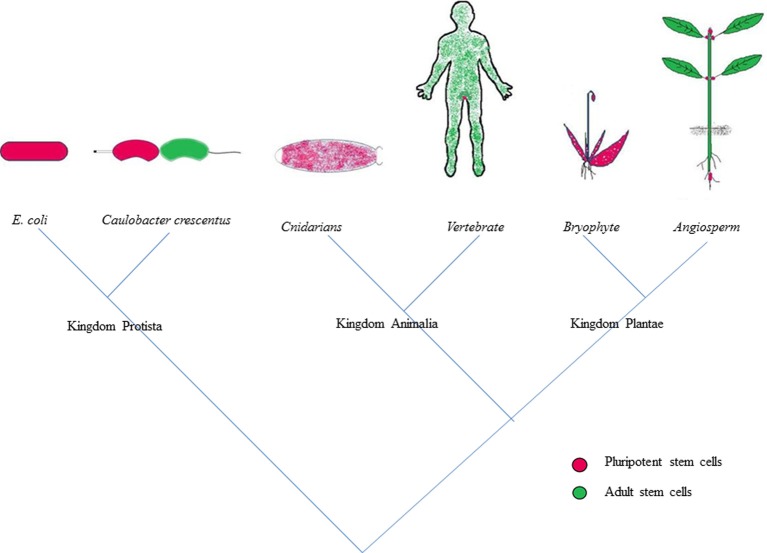
**Tree of regeneration; depicting the competence for regeneration across the kingdoms of life**. Adult stem cells are partly differentiated cells that can give rise to a limited number of cell types while the pluripotent stem cells are competent to give rise to all lineages of cells. The pluripotent stem cells which play a major role in executing the regenerative duties in the lower organisms of each kingdom gave way to adult stem cells and occupy confined niches in higher organisms.

Throughout their lifecycle, plants and animals are subjected to various physical assaults like injury, diseases, or attack by predators. Both these kingdoms deploy the process of regeneration to restore the damage accrued to their body parts as a result of regular wear and tear (Figure 1) (Birnbaum and Sanchez Alvarado, 2008; Sena and Birnbaum, 2010; Sugimoto et al., 2011). Regeneration is highly pronounced in plants compared to animals since they are more prone to abrasion due to their sessile nature (Legendre and Gautheret, 2003). Remarkable regenerative ability is also shown by a wide range of animals. The regeneration of amputated limb by salamanders (Brockes and Kumar, 2002) is the most cited example of regeneration in animals. Hydra exhibits marvelous capability to regenerate the entire body plan by mere reaggregation of its dissociated cells (Gierer et al., 1972). In most cases, the regeneration in plants and animals proceeds *via* the formation of intermediate mass of highly specialized tissue with high regenerative capability called callus and blastema, respectively (Birnbaum and Sanchez Alvarado, 2008). Plants, besides possessing the capability to replace the lost organs, also display a striking ability to regenerate entirely new individuals from the damaged organs, a property unique to plants which was demonstrated in the leaves of begonias and pansies. The plant cells are widely believed to be totipotent as the differentiated cells of the plant possess the capability to give rise to a new plant on provision of suitable *in vitro* culture conditions (Skoog and Miller, 1957; Halperin, 1986). But, the regenerative potential of the cells within the same plant varies with the cell type and also with the different stages of development (Guzzo et al., 1994).

Among the plants, *Arabidopsis thaliana* is one of the most celebrated model systems, which has been extensively exploited for carrying out *in vitro* regeneration studies (Weigel and Glazebrook, 2002). The plant shows high ability to regenerate ablated organs. Laser ablated root and shoot meristem regenerates quickly without any external hormonal supply in *in planta* (Reinhardt et al., 2003; Xu et al., 2006). Similar ability is also shown by other parts of the plant like leaves, bark, cotyledon, and root tip (Pang et al., 2008; Sena et al., 2009; Zhang et al., 2011; Chen et al., 2013). Study of mutants that are defective in regenerating the lost parts gives insight on the molecular pathways involved in providing competence for *in planta* regeneration (Xu et al., 2006; Pang et al., 2008; Sena et al., 2009; Zhang et al., 2012; Chen et al., 2013). Various parts of the plant like root, hypocotyl, cotyledons, leaves, petals, etc., serve as sources of explant for *in vitro* regeneration experiments (Weigel and Glazebrook, 2002). The regenerative fate of the explant is determined by the ratio between two important plant hormones, auxin, and cytokinin (Skoog and Miller, 1957). High auxin to cytokinin ratio destines root fate from the explant, while high cytokinin to auxin ratio specifies shoot regeneration. Regeneration from the explant can proceed either directly or indirectly. The direct mode of regeneration involves the development of either root or shoot directly from the explants (Atta et al., 2009) in contrast to the indirect mode, where an intervening step of callus formation precedes regeneration (Valvekens et al., 1988). Intriguingly, recent body of experiments has brought to lime light, the crucial role played by epigenetic modifications in regeneration (Xu and Huang, 2014).

Studies on various mutant combinations and several marker lines coupled with high resolution live imaging have been carried out to delineate the mechanism of *de novo* organogenesis in *Arabidopsis* (Gordon et al., 2007; Atta et al., 2009; Sugimoto et al., 2010; Chatfield et al., 2013; Motte et al., 2013). Yet many facts pertaining to regeneration like, how cells at the site of damage acquire the competence to regenerate the entire lost body part and what is the initial trigger for reprogramming during regeneration still remains elusive. Detailed aspects of regeneration, the key players involved in *in vitro* regeneration, possible mechanistic insights and the evolutionarily conserved aspects of regeneration across the plant kingdom will be addressed in this review (Figure 1).

## Regeneration upon wounding

**Figure 2:**
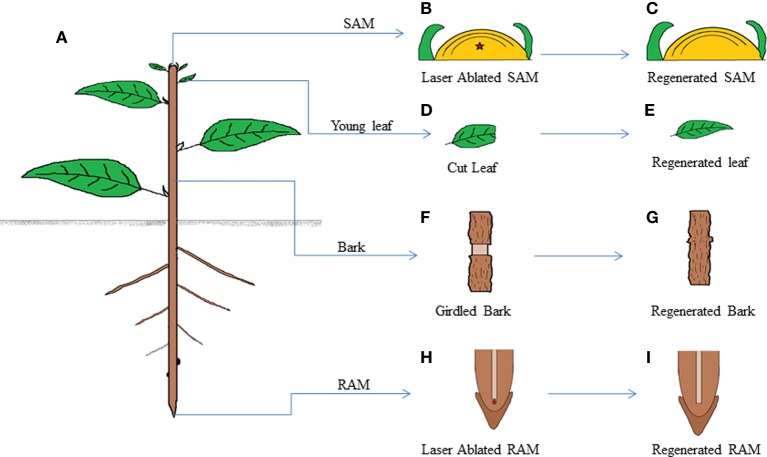
**Schematic drawing showing regeneration ability of various parts of a plant**. A plant shows high levels of regeneration **(A)**. When SAM of the plant is laser ablated, **(B)** the wound gets regenerated without external hormonal application **(C)**. Similarly various plant organs like young leaf **(D,E)**, bark **(F,G)**, and RAM **(H,I)** are able to regenerate the complete organ from the ablated organ without any extraneous hormonal application.

The power to regenerate complex structures at the site of injury has been well celebrated in plants and animals. Limb regeneration in salamander (Godwin et al., 2013; Sandoval-Guzman et al., 2013), tail regeneration in *Xenopus* (Gargioli and Slack, 2004) and fin and heart regeneration in zebra fish (Jopling et al., 2010; Wang et al., 2013) are very good examples for organ regeneration after amputation in animals. Similarly, regeneration of root tip and leaf after excision in *Arabidopsis* (Xu et al., 2006; Sena et al., 2009) and shoot and bark regeneration (Reinhardt et al., 2003; Zhang et al., 2011; Chen et al., 2013) are well documented organ to organ regeneration in plants (Figure 2). The amazing ability of the cells at the injured region to acquire competence to recover precisely the excised part of organ is a long standing area of research for developmental biologists.

**Figure 3:**
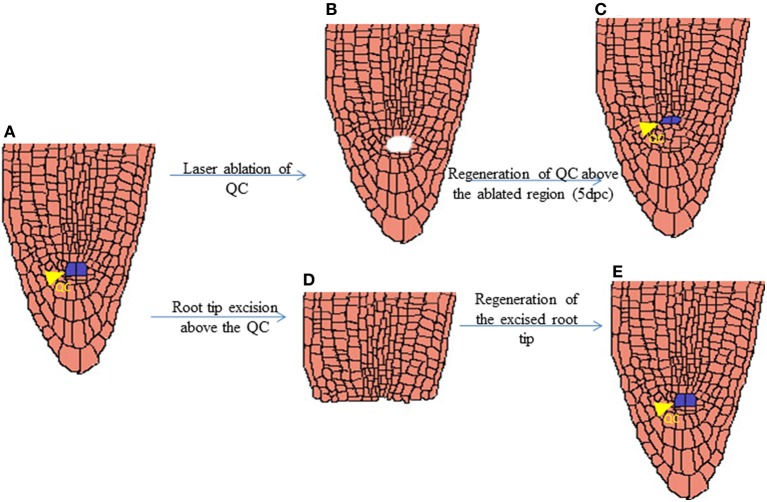
**Schematic representation of regenerated QC after laser ablation and root tip excision**. *Arabidopsis* root tip with Quiescence cells (QC) marked in Blue **(A)**. Root tip after Laser Ablation of QC cells **(B)**. Regenerated QC cells above the ablated region just 5 days post laser ablation **(C)**. Excision of root tip above the QC region **(D)**. Regenerated Root tip **(E)**.

The shoot and root apical meristems are responsible for the indeterminate growth of a plant, therefore the maintenance of these apical meristems during a wound is quite essential for its indeterminate growth. The *Arabidopsis* root meristem exhibits high regenerative potential upon the excision of root tip or laser ablation of quiescent center, QC (Figure 3) (Xu et al., 2006; Sena et al., 2009). Followed by the laser ablation, the auxin distribution at the tip of root meristem gets disrupted which triggers regeneration (Xu et al., 2006). A shift in auxin response is established just after 3 h of QC ablation and a proximally shifted new auxin maxima is developed after 16 h of the ablation, subsequently leading to the respecification of new QC. Interestingly, the change in auxin response induces cell fate changes as evident by the expression of key cell fate regulator, *PLETHORA* (*PLT*) just after 6 h of ablation, i.e., 3 h after the shift in auxin response (Xu et al., 2006). PLT attains a new domain of expression and its activity triggers nuclear localization of SHORTROOT (SHR) in a single layer of provascular cells which in turn induces *SCARECROW* (*SCR*) expression. The new expression domain and activity of *PLT*, *SCR*, and *SHR* genes specify the new QC one or two cell layers above to the ablated QC within about 16 h of ablation. Thereafter the expression and polarization of polar auxin efflux carrier *PINFORMED* (*PIN*) are set facilitating the completion of regeneration (Xu et al., 2006).

Unlike the regeneration of QC after ablation, respecification of lost cell identity in the root meristem after root tip excision does not require maintenance of functional stem cell niche (Sena et al., 2009). While the mutants defective in stem cell maintenance like *scr* and *plt1 plt2* double mutant are recalcitrant for QC regeneration (Xu et al., 2006), the regeneration of cut root meristem does occur in these mutants (Sena et al., 2009). The regeneration of lost cell fate is initiated much before the morphological recovery of stem cell niche of root apical meristem in wild type *Arabidopsis*. This implies that although respecification of QC is an exclusive feature of functional stem cells, the competence for organ regeneration is not unique to stem cells. But it is also distributed among partially differentiated cells as the partially differentiated root meristematic cells retain the competence for organ regeneration.

It is clearly demonstrated that root tip regeneration after excision does not need the activity of *PLT1* and *PLT2* or its downstream targets (Sena et al., 2009). It is important to note that though *plt1 plt2* double mutant cannot maintain root stem cell niche (Aida et al., 2004), the residual stem cell activity retained during initial stages of growth of the seedling helps in the outgrowth of new roots. Necessity of other root *PLT*s such as *PLT3* and *PLT4* which can contribute toward residual stem cell activity in *plt1 plt2* mutant needs to be elucidated. It is possible that the complete removal of root stem cell activity may inhibit regeneration from cut root meristem. Early expression of stem cell markers like *PLT* genes during the regeneration of both root tip and QC is quite intriguing (Xu et al., 2006; Sena et al., 2009). It would be interesting to investigate whether the molecular recovery of stem cell regulators play a critical role for the initiation of the recovery of lost cell fate of the root tip.

Moving up from the root, bark is an important tissue that is prone to physical abrasions and hence the regeneration of bark is of prime importance for the survival of a plant. Significant progress has been made in understanding vascular tissue regeneration after bark girdling in tree species like *Eucommia ulmoides* and *Populus tomentosa* (Pang et al., 2008; Zhang et al., 2011; Chen et al., 2013). Tree plants are able to regenerate new bark and vasculature after wounding. Bark girdling removes phloem and cambium cells, but it leaves differentiating xylem (Zhang et al., 2011). The xylem cells exposed after wounding initiates regeneration by dedifferentiation and form proliferating mass of callus cells (Pang et al., 2008; Zhang et al., 2011). Wound cambium forms from the callus and gives rise to new phloem. Phellogen, which is derived from the wound callus initiates the formation of periderm, the outer layer of the bark, thereby completing the process of bark regeneration. Regeneration of new cell types from xylem cells does not require any external hormone application suggesting initial hormone surge would ramp up in response to wounding. Indeed, accumulating evidence point towards this notion. During the vascular tissue regeneration in *Populus*, the expression pattern of polar auxin transport genes is changed to establish auxin gradient for phloem and cambium recovery (Wang et al., 2009; Zhang et al., 2011). The regenerating cambium expresses the auxin influx carriers *PttLAX2* and *PttLAX3* and auxin efflux carrier *PttPIN1* abundantly in *Populus* (Zhang et al., 2011). Xylem cells acquire competence to regenerate by epigenetic changes and cell cycle re-entry in *Populus* (Zhang et al., 2011). At the initial point of regeneration the xylem specific genes get downregulated while phloem and cambium specific genes are upregulated in the regenerating tissues (Zhang et al., 2011). The activation of phloem specific genes such as *ALTERED PHLOEM DEVELOPMENT* (*APL*), *KANADI* (*KAN*), and *DNA binding with one finger* (*Dof)* and cambium specific genes such as *CLAVATA1* (*CLV1*), *CLV2*, *AINTEGUMENTA1* (*ANT1*), *SHR*, and *SCR* promote the repatterning of hormone distribution in the regenerating tissues (Zhang et al., 2011). Simultaneously the cell signaling network is re-established to complete the process of vascular tissue regeneration.

Reinhardt et al. (2003) laser ablated the organizing center (OC) at the shoot apical meristem (SAM) of tomato, similar to the ablation of quiescent center of *Arabidopsis* root performed by Xu et al. (2006). The ablation of SAM however did not cause any significant patterning defects nor did affect the number of primordia that emerged from the meristem. As in root, the regeneration of meristem started immediately after the wounding of OC. The laser ablation of OC caused the complete disappearance of the meristem organizer *LeWUS*, tomato *WUS* homolog (Reinhardt et al., 2003). Ectopic expression of *LeWUS* as a ring around the ablated region is noticed within 1 day after the ablation. This is strikingly similar to the reappearance of *WOX5* after laser ablation of QC in *Arabidopsis* root. The *LeWUS* levels accumulated to one locus, or in rare cases to two around the ablated region after 2 days reinstating the functional meristem expressing *LeWUS* (Reinhardt et al., 2003). Another striking feature of the laser ablation experiments conducted on the shoot meristem is the inability of cells at L1 layer of the shoot meristem to regenerate. When a few cells in L1 layer were laser ablated, they were unable to repair the wound (Reinhardt et al., 2003). This is quite different from the mode of regeneration seen in root where the incisions made on the root even above the QC are repairable (Sena et al., 2009).

What is the initial trigger for regeneration? It is a fundamental question asked by anyone working on regeneration. Auxin plays an instrumental role in the positioning of root stem cell niche (Aida et al., 2004; Cheng et al., 2013) and also in shoot and root organogenesis (Benkova et al., 2003). Auxin response is the earliest key determinant for the initiation of respecification of QC (Xu et al., 2006). The mutants defective in QC regeneration like *scr*, *shr*, and *plt1 plt2* double mutant cannot maintain new auxin maxima after the laser ablation of QC suggesting cell fate specification occurs prior to high auxin response build up which in turn leads to completion of regeneration.

Importance of polar auxin transport is evident from the experiment where regeneration at root meristem failed to occur when the auxin transport was blocked by using N-1-naphthylphthalamic acid (NPA) (Sena et al., 2009). Also, many genes were induced just after the excision of root tip in response to the aberrant levels of auxin (Sena et al., 2009). In light of these experiments, we reinforce the notion that the initial trigger for regeneration is the changes in auxin response. In shoot meristem, the levels of auxin and cytokinin are kept at a constant level by mutual inhibition. The disruption of auxin transport by *PIN1* mutation causes patterning defects by preventing the emergence of primordia. However, the patterning of the shoot primordia is not affected during the ablation, suggesting that the activity of *PIN1* mediated auxin transport is not compromised during the ablation. Hence studying the regeneration of ablated shoot in mutants defective in auxin transport will shed light on the role of auxin in shoot meristem regeneration.

Another question would be to find the initial cell triggering regeneration. Does the competence for regeneration vary according to cell types? During the repatterning of excised root meristem, all cell types constituting the root tip are involved in regeneration (Sena et al., 2009). The competence to regenerate is not a unique feature of a particular cell type, but instead a common feature for many cell types atleast in the meristematic zone. A similar scenario is found in salamander limb regeneration, where the cells at the site of injury keep a memory of their tissue origin and facilitate for tissue specific proliferation (Kragl and Tanaka, 2009). The regeneration competence is not similar at all developmental stages of differentiated cells. While a high competence for regeneration is retained till 130 μm from the root tip, the proficiency of regeneration gradually drops at the proximal end of meristematic region where the cells start entering into the differentiation zone (Sena et al., 2009). The high regeneration potential of young leaves of *Arabidopsis* and recalcitrance towards regeneration in adult leaves (Sena et al., 2009) reiterates the thought that the competence to regenerate is highly dependent on the developmental stages of cells (Figure 2). The leaf regeneration also confirms that the regeneration potential is distributed among cells which are at their early developmental stages. Unlike root tip and leaf regeneration where the repatterning initiated directly from young differentiating cells without callus formation, the vascular tissue regeneration after bark removal involves the dedifferentiation of adult differentiated xylem cells to pluripotent callus (Zhang et al., 2011). Taken together, the competence to regenerate is conferred onto most of the cell types at their young developmental stages.

## *De novo* organogenesis

The ability of plants to regenerate wounded organs is important for their survival. But for studying the mechanisms involved in regeneration and exploiting the potentials of plant regeneration, *in vitro* culturing of plant tissue is required. The cells thus cultured are competent to form different organs of an organism when proper developmental cues are provided. When germline stem cells and induced pluripotent stem cells provides an excellent system for studying *de novo* organogenesis in mammalian system (Donovan and Gearhart, 2001), similar induction of potency for regeneration can be achieved in plants by treating them on high auxin media (Gordon et al., 2007). The callus thus obtained is generally competent to regenerate shoot and an entire plant when treated with proper hormonal levels (Skoog, 1950; Skoog and Miller, 1957; Gordon et al., 2007).

Incubating various explants on auxin rich callus inducing medium (CIM) induces the formation of callus. Callus thus obtained was thought to be dedifferentiated tissue, but recent studies indicate that they are well organized and resemble root (Sugimoto et al., 2010). Thus, they appear to be partly differentiated tissue. Several lines of evidence point toward this notion as callus derived from various explants display gene expresssion profile similar to lateral root (Sugimoto et al., 2010). Many root specific genes like *WOX5, SCR, SHR, PLT1*, and *RCH1* are expressed in calli obtained from both aerial and ground explants (Table 1) (Atta et al., 2009; Sugimoto et al., 2010).

**Table 1:** **List of key genes involved in *de novo* organ regeneration process**.

**Gene**	**Role during callus formation**	**Role during *de novo* shoot regeneration**	**References**
*ALF4* (*ABERRANT LATERAL ROOT FORMATION 4*)	No callus formation in *alf4* mutant	Not described	Sugimoto et al., 2010
*ARR5* (*ARABIDOPSIS RESPONSE REGULATOR 5*)	Upregulated at later stages of callus formation	Expressed in developing shoot meristem but absent in organ primordia	Gordon et al., 2007; Atta et al., 2009
*CLV3* (*CLAVATA3*)	Slightly expressed upon incubation on CIM	Upregulated in developing shoot meristem	Gordon et al., 2007; Atta et al., 2009
*CUC* (*CUP SHAPED COTYLEDON*)	Upregulated	*CUC2* is upregulated in developing shoot meristem and organ primordia Ectopic over-expression of *CUC1/CUC2* can enhance *de novo* shoot formation on SIM. Shoot regeneration efficiency is reduced in *cuc1cuc2* double mutant	Cary et al., 2002; Daimon et al., 2003; Gordon et al., 2007
*ESR* (*ENHANCER OF SHOOT REGENERATION*)	*ESR1* induced upon incubation on CIM	*ESR1* transiently gets upregulated soon after transfer onto SIM and declined after 2 days. Over expression of *ESR1* or *ESR2* enhances *de novo* shoot regeneration in presence of cytokinin. Reduced shoot regeneration in single and double mutants	Banno et al., 2001; Ikeda et al., 2006; Matsuo et al., 2011
*LBD* (*LATERAL ORGAN BOUNDARIES DOMAIN*)	Overexpression triggered callus formation on hormone free medium and loss of function caused suppression of callus formation	Not described	Fan et al., 2012
*PIN1* (*PIN FORMED 1*)	Upregulated at early stages of callus formation but gets downregulated later	Accumulated in developing shoot meristem and organ primordia In *pin1-4* mutant, shoot regeneration decreased to 20% of wild type	Gordon et al., 2007; Atta et al., 2009
*PLT1* (*PLETHORA1*)	Upregulated	Downregulated	Atta et al., 2009; Sugimoto et al., 2010
*SCR* (*SCARECROW)*	Upregulated	Not described	Sugimoto et al., 2010
*SHR* (*SHORT-ROOT*)	Upregulated	Not described	Sugimoto et al., 2010
*STM* (*SHOOT MERISTEMLESS*) stm-1	Rarely expressed upon incubation on CIM	Upregulated in developing shoot meristem In *stm-1* mutant, shoot regeneration decreased to 15% of wild type	Barton and Poethig, 1993; Gordon et al., 2007; Atta et al., 2009
*RCH1* (*ROOT CLAVATA-HOMOLOG1*)	Upregulated	Downregulated	Atta et al., 2009
*WOX 5* (*WUSCHEL-RELATED HOMEOBOX 5)*	Upregulated	Not described	Sugimoto et al., 2010
*WUS* (*WUSCHEL*)	Not expressed in callus	Upregulated during direct/indirect shoot regeneration in LRP/callus on SIM In strong *wus-1* mutant, shoot regeneration reduced to 5% of wild type. Ectopic overexpression of *WUS* makes *de novo* shoots/somatic embryos on hormone free medium	Zuo et al., 2002; Gallois et al., 2004; Gordon et al., 2007; Atta et al., 2009; Chatfield et al., 2013

All plant organs like shoot, leaves and auxiliary branches arise from the pool of meristematic cells already present in apical meristems and axillary buds. However, lateral root emerges out of an already differentiated cell lineage, the xylem pericycle cells (Gordon et al., 2007; Atta et al., 2009). The emergence of meristematic cells from a lineage of differentiated cells is intriguing and provides an excellent model on how pluripotency can be achieved from differentiated cells (Takahashi and Yamanaka, 2006; Yu et al., 2007). The emergence of lateral root at any location on a root is signaled by the establishment of local auxin maxima and activation of auxin responses in the pericycle cells (Celenza et al., 1995; Casimiro et al., 2001). Similarly during callus induction, the auxin rich medium provided externally increases the auxin levels in explants. As both callus induction and lateral root primordium (LRP) initiation is mediated by high auxin conditions (Che et al., 2007), it is fair to speculate that callus emergence from pericycle cells is similar to LRP formation. Furthermore, *aberrant lateral root formation4* (*alf4*) mutant which blocks the initial division of pericycle cells and hence is defective in lateral root initiation (Celenza et al., 1995) completely abolishes callus formation upon CIM treatment (Sugimoto et al., 2010). However, abscisic acid (ABA) treatment of the wildtype explant, which does not prevent lateral root initiation, but prevents its outgrowth is able to form callus (Sugimoto et al., 2010). This suggests that callus development is directly related to the initial development of LRP. Intriguingly the callus formation from the aerial parts of *alf4* mutant is also abrogated, suggesting that pericycle-like cells are distributed throughout the plant body and are required for the callus formation (Sugimoto et al., 2010). Several lines of evidence support the notion that pericycle or pericycle-like cells are involved in callus formation irrespective of the context of explants used for regeneration. Sugimoto et al. (2010) analyzed the expression pattern of pericycle specific marker J0121 in aerial parts like leaves and hypocotyl to confirm the presence of pericycle like cells. They found the signal to be enriched around the midvein of cotyledon and leaf and also around the vasculature. Treating the aerial and ground explants on CIM, the area of expression of J0121 get enhanced and once the outgrowth starts to attain its identity, the expression level is reduced. Further, overexpression of four *LATERAL ORGAN BOUNDARIES DOMAIN (LBD)* genes *viz*. *LBD16, LBD17, LBD18*, and *LBD29* triggers callus formation without external hormonal supplement (Fan et al., 2012). But, when these *LBD* genes are suppressed, lateral root formation is impaired and no callus is formed even upon CIM treatment (Fan et al., 2012).

**Figure 4:**
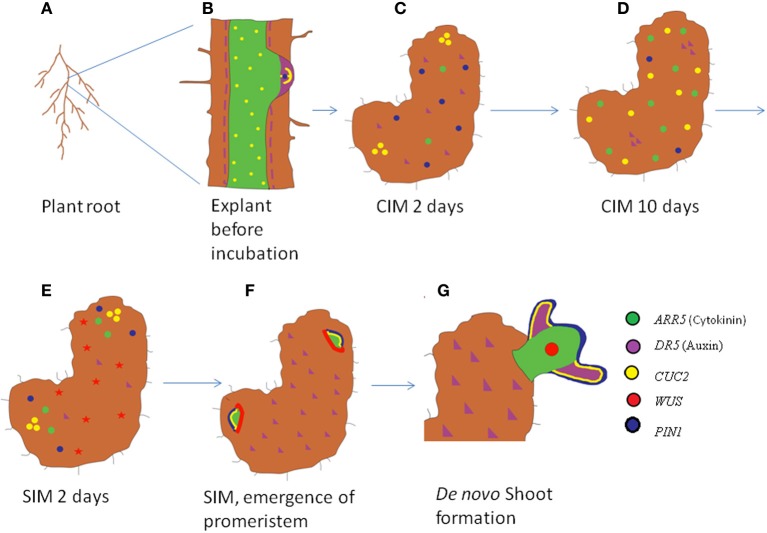
**Schematic of expression dynamics of regulatory genes during *de novo* shoot organogenesis**. The root explant of *Arabidopsis* **(A,B)** with an emerging lateral root expresses auxin responsive *DR5* at the tip of the LRP (purple) coinciding with the expression domain of *PIN1* (magenta), cytokinin responsive *ARR5* (green) in LRP and root stele and *CUC2* (yellow) in a subset of cells in lateral root meristem and root stele **(B)**. The expression domain of auxin and *PIN1* expands in the proliferating callus 2 days after CIM treatment, while *CUC2* gets accumulated in patches and cytokinin level start rising **(C)**. Ten days after CIM induction, *CUC2* and ARR5 expression is seen throughout the callus while *PIN1* and *DR5* are weakly expressed in callus **(D)**. Two days post SIM induction, *CUC2*, and *ARR5* get confined to patches while *WUS* expression is induced throughout the callus **(E)**. Regions of high *CUC2* and *ARR5* expression marked the emerging shoot promeristem while *WUS* is expressed in the peripheral cells. *DR5* expression is low at the area of shoot meristem emergence **(F)**. Once the *de novo* shoot emerges out, *WUS* occupies the central niche with high auxin and *CUC2* levels are confined to primordial **(G)**.

**Figure 5:**
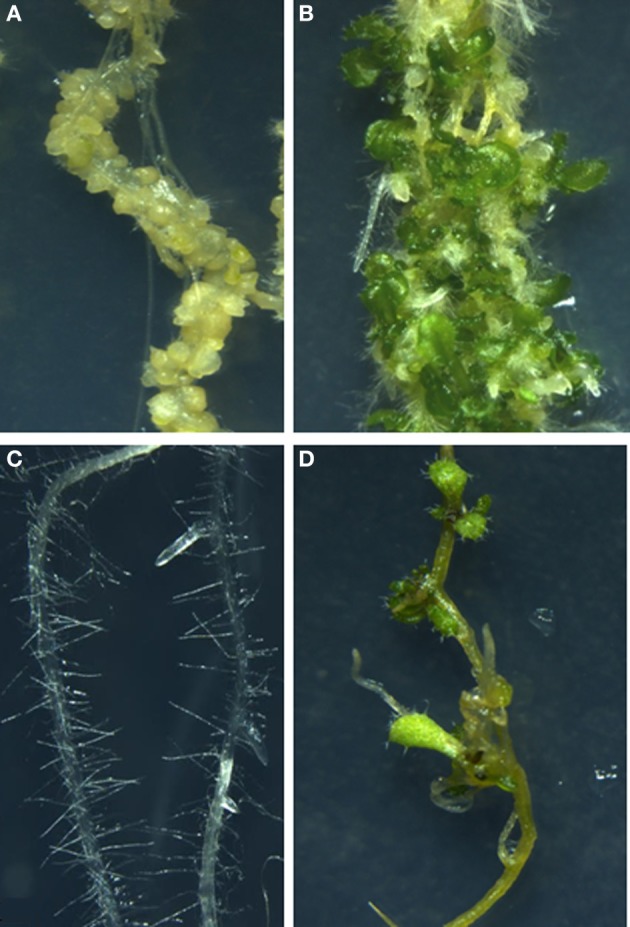
**Direct and Indirect *de novo* organogenesis**. Callus formation from *Arabidopsis* root explant on auxin rich callus inducing medium (CIM) **(A)**. *De novo* shoot regeneration on callus incubated on cytokinin rich shoot inducing medium (SIM) **(B)**. *Arabidopsis* root explant with lateral root primordia from where lateral root emerges out incubated on normal MS medium **(C)**. Direct shoot regeneration from lateral root primordia of *Arabidopsis* root upon incubation on cytokinin rich medium **(D)**.

Interestingly treatment of explants with diphtheria toxin chain A (DTA) specifically ablates xylem-pole adjacent pericycle cell files (Laplaze et al., 2005) and this abrogates lateral root formation and further blocks callus induction on CIM (Che et al., 2007). Collectively all these evidences hints us that the competence for callus formation is largely influenced by the ability of the plant to initiate lateral roots from the pericycle cells (Laplaze et al., 2005; Atta et al., 2009). Irrespective of the explants used for callus induction, callus arises from pericycle or pericycle like cells and competence for callus induction is provided by pericycle specific genes (Figure 5) (Sugimoto et al., 2010). The transformation of pericycle cells to organogenic callus occurs pretty rapidly and the competence is maintained only for a specific time window. The competence for organogenesis is acquired by incubation of explants on CIM for a minimum of 48 h. Within this period, PIN1 marks the callus outgrowth (Gordon et al., 2007). Subsequently the auxin response exhibited by *DR5* promoter spikes to a maximum. Further incubation on CIM leads to the decrease in the domain and expression levels of both *PIN1* and auxin response. As their levels go down, the expression levels of cytokinin responsive *ARABIDOPSIS RESPONSE REGULATOR 5* (*ARR5*) and boundary genes *CUP SHAPED COTYLEDON 1*(*CUC1*) and *CUC2* increases and gets localized to the proliferating callus (Figure 4) (Gordon et al., 2007). The callus is competent to form organs like shoot and root during these stages. But the competence thus obtained is transient. After incubation of the callus in CIM for more than 14 days, the competence to switch to shoot fate is rapidly reduced and the callus gets differentiated completely to root fate (Christianson and Warnick, 1983; Gordon et al., 2007).

The next hurdle for *de novo* organogenesis is the patterning of pluripotent callus to specific organs. It took 19 years after the discovery of callus inducing medium for finding the proper medium for converting the callus to desired organ fates. Skoog and Miller (1957) discovered that high cytokinin to auxin ratio induces shoot regeneration from callus and high auxin concentration leads to formation of root from the medium (Skoog and Miller, 1957). The induction of root from callus is easier and trivial because the callus itself is having root identity (Figure 4). Therefore, the root induction needed the incubation on root inducing medium (RIM) which is rich in auxin.

But, shoot organogenesis is comparatively complex as new set of genes need to be turned on. Some of the genes expressed in callus get spatially regulated and several key regulatory interactions get established to switch the root fate to shoot (Gordon et al., 2007; Atta et al., 2009). Just after the incubation on cytokinin rich shoot inducing media (SIM), the callus which was expressing root specific genes all throughout undergoes a morphosis to a mass of cells that is having a localized expression of shoot specific genes (Figure 4). The SIM induction of calli causes partitioning of cell fates evident by differential expression of different markers (Gordon et al., 2007). It induces the spatial and temporal localization of shoot patterning genes like *CUC2* and expression of shoot meristem regulators like *WUS* (Gordon et al., 2007). The auxin responsive *DR5* expression also gets localized to the regions of callus that did not initiate shoot while cytokinin responsive *ARR5* and polar auxin transporter *PIN1* gets confined to those areas of callus that is low on *DR5* expression (Gordon et al., 2007; Atta et al., 2009). Simultaneous to *PIN1* expression, *ARABIDOPSIS THALIANA MERISTEM L1 LAYER* (*ATML1*) gets upregulated in the superficial layer of shoot promeristem. Consequently, the expression domain of *WUS* is confined to the center of the promeristem and another important shoot meristem regulator, *SHOOT MERISTEMLESS* (*STM*) is accumulated in a ring of surrounding cells within the promeristem, meanwhile the *CUC2* expression is delocalized to the site of shoot primordia formation (Figure 4) (Gordon et al., 2007). Subsequently *CLAVATA3* (*CLV3*), stem cell marker, is expressed in the central zone of shoot meristem just above to the *WUS* expression domain, where the stem cell niche resides. As shoot primordia are initiated from the peripheral zone of the shoot meristem, *PIN1* gets upregulated in the shoot primordia (Table 1) (Gordon et al., 2007).

The callus formed upon hormonal treatment has great resemblance to LRP. Hence it is expected that the LRP should give rise to *de novo* organs without the formation of callus. The direct regeneration of shoot from LRP when treated with cytokinin rich media (Atta et al., 2009; Chatfield et al., 2013) suggests that shoot regeneration do not always need callus formation, but the LRP cells are competent enough to trigger the shoot fate directly (Figure 5). The direct shoot regeneration from pericycle cells adjacent to protoxylem, which form the LRP, suggests that the pericycle cells are highly flexible to acquire different cell fate than only converting itself to root-like callus cells. Immediately after cytokinin treatment the gene expression profile of LRP changes and it expresses shoot meristem markers like *WUS, CLV3*, and *STM* that are not normally expressed in root (Atta et al., 2009; Chatfield et al., 2013). Changes in auxin gradient and polar localization of *PIN1*, together with the dynamic expression of shoot meristem regulators define shoot meristem formation from LRP in 5–6 days of induction on cytokinin rich medium (Atta et al., 2009). Interestingly, ectopic expression of *WUS* is sufficient to initiate *de novo* shoot formation directly from *Arabidopsis* root (Gallois et al., 2004). The mis-expression of *WUS* in the root initiates to change the entire gene expression profile of root into shoot specific. Taken together, the growing body of evidences suggests that the pericycle cells have amazing regenerative ability and it can be converted into lateral root, shoot, or callus depending upon the different hormonal treatment.

*De novo* organogenesis can be obtained either directly from root explants, or through an intervening callus stage. But for the regeneration to occur the presence of competent pericycle cells that can initiate LRP is required. When LRPs act as the initiation point in direct regeneration, presence of comptent pericycle is required for callus formation. The callus which has a LRP identity when incubated on cytokinin rich media undergoes a morphosis and expresses shoot meristem regulators. Not surprisingly, mutation in shoot meristem regulators like *CUC2, WUS, STM*, and *PIN1* display decrease in *de novo* shoot formation. However, mutation in these genes do not abolish the *de novo* shoot initiation completely (Barton and Poethig, 1993; Daimon et al., 2003; Gordon et al., 2007). Future studies should unravel additional key regulators that provide competence for *de novo* organogenesis from callus.

### microRNA mediated regulation during *de novo* shoot regeneration

Recent studies on microRNAs (miRNAs) show their role in *de novo* organogenesis. These small endogenous non-coding RNAs (21–24 nucleotide length) that regulate gene expression at transcriptional and post-transcriptional level in plants and animals are implicated to have crucial roles in development (Bartel, 2004). In addition to the regulation of developmental processes like embryogenesis, meristem organization, leaf polarity, and vascular development (Jung and Park, 2007; Liu et al., 2007), they are involved in cell proliferation and differentiation. In plants, many miRNAs are differentially regulated during callus formation and *de novo* shoot formation (Luo et al., 2006; Chen et al., 2011; Qiao et al., 2012; Liu et al., 2013). A unique set of miRNAs are differentially expressed during embryogenic callus formation in rice (Luo et al., 2006; Chen et al., 2011). Similarly, a group of miRNAs which are significantly abundant during callus formation and *de novo* shoot regeneration in *Arabidopsis* has been identified (Qiao et al., 2012). The differential expression of few miRNAs in totipotent and non-totipotent calli reveals their role in cell proliferation and differentiation. One of such miRNA is miR160 which is scarce in totipotent calli but abundant in non-totipotent calli (Qiao et al., 2012). Interestingly miR160 negatively controls the expression of *AUXIN RESPONSE FACTOR10* (*ARF10*) gene during *de novo* shoot regeneration (Qiao et al., 2012). miR160 mediated regulation of ARF10 is also pronounced during seed germination and post embryonic development in *Arabidopsis* (Liu et al., 2007). Overexpression of miR160 leads to reduction in shoot regeneration. Meanwhile the miR160 resistant form of ARF10, mARF10, shows high regeneration potential (Qiao et al., 2012). Other miRNAs like miR165/166 are involved in the regulation of shoot meristem maintenance, leaf polarity and floral development in *in planta* (Jung and Park, 2007). Intriguingly, these two miRNAs are also involved in shoot regeneration in *Arabidopsis* in parallel to *WUS/CLV3* and *REV* pathways (Liu et al., 2013). It has been shown that miR164 controls the dynamic expression of *CUC2* during the organ initiation in *in planta* (Peaucelle et al., 2007). But the expression pattern of CUC2 remained the same in both transcriptional and translational fusion during early stages of *de novo* shoot regeneration (Gordon et al., 2007). So, whether miR164 regulates CUC2 expression during *de novo* organogenesis is yet to be elucidated. Although efforts have been put to identify some of the miRNAs during callus formation and shoot regeneration, a complete picture of miRNA regulated gene expression during *de novo* shoot regeneration is not clear and hence a comprehensive study is demanded in this direction. Together, the growing body of evidences suggest that miRNA mediated post-transcriptional regulation is crucial for the cells to maintain pluripotency and to differentiate and give rise to lateral organs during *in vitro* culture.

## Somatic embryogenesis

Somatic embryogenesis can be an attractive tool to study the developmental events during zygotic embryogenesis particularly when the zygotic embryos are not easily accessible for carrying out biochemical and molecular analysis. Somatic embryos are defined as structures that are having a somatic cell origin but share the morphology and anatomy of zygotic embryo (Bassuner et al., 2007; Birnbaum and Sanchez Alvarado, 2008; Gliwicka et al., 2013). During somatic embryogenesis, differentiated cells reverse their developmental program and acquire embryonic potential (El Ouakfaoui et al., 2010; Gliwicka et al., 2013). Though somatic embryo and zygotic embryo arise from different lineages of cell, the developmental processes involved are mostly conserved (Zimmerman, 1993; Jimenez and Bangerth, 2001; Birnbaum and Sanchez Alvarado, 2008; Bai et al., 2013). Like the zygotic embryo, somatic embryo proceeds from globular stage to torpedo stage and has many morphological similarities to the stages of zygotic embryogenesis (Meinke, 1991; Zimmerman, 1993; Su et al., 2009). Somatic embryogenesis from somatic cells is achieved by incubating the explants on medium containing specific amount of synthetic auxin 2, 4-dichlorophenoxyacetic acid (2, 4-D) and subsequent transfer to auxin free medium (Sugiyama, 2000; Mordhorst et al., 2002). Somatic embryo can be easily distinguished from other structures like adventitious shoots and fused shoots which are formed during the induction of a somatic embryo by its bipolar nature (shoot and root pole). The bipolar nature is evident in a somatic embryo by the localized expression of *WUS* and *CLV3* in the shoot meristem of the heart shaped stage (Su et al., 2009) and *PIN4* expression and high *DR5* activity in its root meristem (Bassuner et al., 2007). While the adventitious and fused shoot remains attached to the explant and does not develop an anatomically recognizable root meristem, the somatic embryo which arises of a small cell cluster detaches fully or just has a thin connection with the callus and has a distinguishable shoot and root (Pillon et al., 1996; Mordhorst et al., 2002; Gaj et al., 2005; Bassuner et al., 2007). Other features of the somatic embryos include the absence of trichomes in the first pair of leaves (cotyledons), thick cell wall, big nucleus, and single nucleolus.

Like zygotic embryogenesis, a complex and similar set of regulatory interactions are involved during somatic embryogenesis which provide the necessary information to acquire new fate and for further development. Unraveling the molecular mechanism governing the somatic embryogenesis is important to improve the existing regeneration protocol. The role played by some of the key players of somatic embryogenesis such as *WUS* (Zuo et al., 2002)*, AGAMOUS LIKE15* (*AGL15*) (Harding et al., 2003)*, BABY BOOM* (*BBM*) (Boutilier et al., 2002; El Ouakfaoui et al., 2010)*, EMBRYOMAKER* (*EMK/AIL5*) (Tsuwamoto et al., 2010), and *LEAFY COTYLEDON* (*LEC*) (Gaj et al., 2005; Guo et al., 2013) have been recently elucidated. Ectopic overexpression of *WUS*, a homeodomain transcription factor, induces somatic embryos in the root and shoot apices of intact seedlings as well as root explants of *Arabidopsis* on hormone free medium (Zuo et al., 2002). Even when *Arabidopsis AtWUS* gene is overexpressed in cotton plants, it triggers somatic embryogenesis (Bouchabke-Coussa et al., 2013) signaling that the developmental processes involved in somatic embryogenesis may be conserved across species. Similarly, members of the *AP2* family of transcription factors such as *EMK/AIL5* (Tsuwamoto et al., 2010) and *BBM* (Boutilier et al., 2002; El Ouakfaoui et al., 2010) have the capacity to induce somatic embryogenesis when ectopically expressed. In *BBM* or *AIL5* overexpressed seedlings, somatic embryos are formed on the cotyledon, leaf, petiole, and shoot apex (Boutilier et al., 2002; El Ouakfaoui et al., 2010; Tsuwamoto et al., 2010). During the somatic embryogenesis in soybean plants, the mRNA levels of *GmPLT2* and *GmAIL5*, homologs of *Arabidopsis PLT2* and *AIL5*, respectively, have been shown to be upregulated (El Ouakfaoui et al., 2010).

The levels of both exogenous and endogenous auxin play an important role during somatic embryo formation (Su et al., 2009; Bai et al., 2013; Wojcikowska et al., 2013). The process of somatic embryogenesis requires appropriate levels of external auxin supply (Wojcikowska et al., 2013). Initially when the somatic explant is treated with 2,4-D rich medium, embryonic callus is induced. The increased level of exogenous auxin enhances ethylene biosynthesis by activating 1-aminocyclopropane-1-carboxylate synthases (ACSs) (Abel et al., 1995; Tsuchisaka and Theologis, 2004; Bai et al., 2013). Thus, an increased level of ethylene is maintained in the embryonic callus. The ethylene thus induced helps in auxin transport and regulates asymmetric auxin distribution by inhibiting auxin biosynthetic *YUCCA* (*YUC*) genes (Muday et al., 2012). When this callus is treated with hormone free medium, due to the absence of external auxin, ethylene levels start to diminish (Bai et al., 2013). Thereby the control on endogenous auxin synthesis is lifted and the endogenous expression of *YUC* genes is reinstated. *LEC2* is a seed specific gene (Santos-Mendoza et al., 2005) and is a key regulator of embryo and seed development (Braybrook and Harada, 2008) which gets upregulated during somatic embryogenesis. It is epigenetically regulated by *PICKLE* (*PKL*) and *FERTILIZATION INDEPENDENT ENDOSPERM* (*FIE)* (Ogas et al., 1999; Bouyer et al., 2011) during other developmental stages. The activation of *LEC2* also helps in increasing the levels of endogenous auxin synthesis indirectly. The polarization of PIN1 within 16 h of induction along with the *LECs* and ethylene plays an important role in setting the auxin gradient (Liu et al., 1993; Su et al., 2009). During these initial stages of somatic embryogenesis, WUS and PIN1 occupy the areas in the callus where the amount of auxin is less (Su et al., 2009). After 36 h of induction, WUS and PIN1 get co-localized in somatic pro-embryo which further proceed for the completion of the process of somatic embryogenesis (Su et al., 2009). Treatment of embryonic calli with auxin transport inhibitor N-1-naphthylphthalmic acid (NPA) causes the suppression of *WUS* induction and the inhibition of somatic embryogenesis. Thus, establishment of auxin gradients and polar localization of PIN1 somehow regulate the expression of *WUS* in the embryonic calli and the cumulative effort of auxin gradient and *WUS* expression lead to the formation of somatic embryos (Su et al., 2009). Later, the stem cells of the embryo are marked by a non-overlapping expression of *CLV3* (Su et al., 2009). Meanwhile *LEC2* which got upregulated in the initial stages of somatic embryogenesis, starts accumulating in cotyledon primordia of the emerging embryo. CUC2 also follows a localization and expression pattern of *LEC2* and gets compartmentalized to the cotyledon boundary and the STM levels increase till the proper somatic embryo is defined (Su et al., 2009).

Once the initial competence is set by treatment of explants with auxin rich media, rest of the processes involved in somatic embryogenesis follows up like a cascade. Gao et al. (2008) have shown that auxin can act as a self-organizing signal to direct polar auxin transport and to establish auxin gradient. It is quite reasonable to think that the same self-organizing signal is active to establish the initial auxin gradient during the early stages of somatic embryogenesis and regeneration. Once this signal is set, it promotes the polar localization of PIN1 and leads to the formation of somatic embryo. This auxin gradient initiates the process of regeneration. But the processes involved in setting the initial gradient of auxin that give competence for somatic embryogenesis remain largely unknown. Taken together, the molecular pathways and hormonal controls involved in *de novo* organogenesis are similar, both these processes end up forming two distinct structures. When somatic embryogenesis reflects the totipotency of the cell, *de novo* organogenesis presents its pluripotency. Further studies on the cumulative roles of plant hormones like cytokinin and ethylene and control on the endogenous levels of auxin by LEC and polar auxin transport will shed light on the early processes involved in setting the competence for somatic embryogenesis.

## Epigenetic modifications during regeneration

Chromatin remodeling is the principal phenomenon underlying reprogramming that occurs during regeneration. The fate of the cells at the site of damage is switched to adopt a new fate in the process of regenerating the lost organ. It is accomplished by reorganization of the gene expression pattern, brought about by various epigenetic mechanisms (Barrero and Izpisua Belmonte, 2011). The epigenetic mechanisms that operate in both plants and animals are similar in terms of their function and mode of action. DNA methylation and post-transcriptional modifications of N-terminal tails of core histone proteins such as acetylation, methylation, ubiquitination, phosphorylation, and ADP ribosylation, occurring primarily at the upstream regulatory sequences of specific genes are the major contributing mechanisms to chromatin remodeling (Jenuwein and Allis, 2001; Richards and Elgin, 2002). DNA methylation, histone deacetylation and histone methylation at specific amino acid residues such as lysine9 of histone3 (H3K9), lysine27 of histone3 (H3K27), lysine20 of histone4 (H4K20) negatively regulate the gene expression, while histone acetylation, DNA hypomethylation and histone methylation, specifically at lysine4 of histone3 (H3K4), lysine36 of histone3 (H3K36) and lysine79 of histone3 (H3K79) work as positive switch of gene expression (Wade et al., 1997; Jenuwein and Allis, 2001; Lusser et al., 2001; Zhang and Reinberg, 2001; Richards and Elgin, 2002). The epigenetic regulators act by modifying the structure of chromatin, which in turn alter the accessibility of chromatin to the transcriptional machinery resulting in the modulation of gene expression (Callinan and Feinberg, 2006). Transcriptional repression *via* H3K27 methylation is mediated by Polycomb group (PcG) of proteins. PcG proteins are highly conserved proteins and play a pivotal role in long-term gene silencing. The PcG proteins form two distinct protein complexes, Polycomb repressive complex1 (PRC1) and PRC2 (Francis and Kingston, 2001; Saurin et al., 2001; Cao et al., 2002; Czermin et al., 2002; Kuzmichev et al., 2002; Muller et al., 2002). While PRC2 is involved in leaving an epigenetic signature by trimethylating H3K27 (Cao et al., 2002; Czermin et al., 2002; Kuzmichev et al., 2002; Muller et al., 2002), PRC1 complex stabilizes the methylation mark via ubiquitination of lysine 119 and lysine 121 on H2A, in animals and plants, respectively. This causes localized heterochromatin formation, resulting in gene repression (Schuettengruber and Cavalli, 2009).

**Figure 6:**
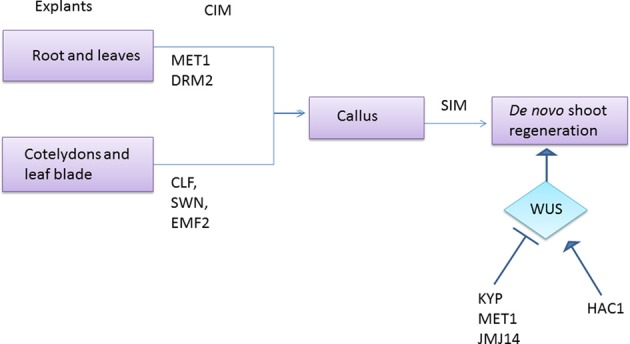
**Role of epigenetic modifiers in *de novo* shoot formation**. Explants from various parts of the plant are having different epigenetic modifiers to control gene expression during callus formation. When *MET1* and *DRM2* are involved in the callus formation from root and leaves, *CLF*, *SWN*, and *EMF2* does the same in explants derived from cotyledon and leaf blade. Further control on shoot organogenesis is achieved by monitoring the expression of *WUS*, which is epigenetically inhibited by KYP, *MET1*, and *JMJ14* and is enhanced by *HAC1*.

In *Arabidopsis*, the PRC2 proteins, CURLY LEAF (CLF), SWINGER (SWN), and EMBRYONIC FLOWER2 (EMF2) are implicated to play a major role in the formation of callus. The redundant proteins, CLF and SWN are homologous to Enhancer of Zeste, E(z) (Chanvivattana et al., 2004), while EMF2 is homologous to Suppressor of Zeste 12 (Su(z)12) of *Drosophila* (Yoshida et al., 2001). *clf swn* double mutant and *emf2* mutant, are defective in callus formation, from cotyledons and leaf blade but not from root (He et al., 2012). This suggests the plausible role of PRC2 components in suppressing the leaf-specific regulatory genes, which is essential for the transition of leaves into pluripotent callus (Figure 6). Recent pioneering work on root and leaf explants have shown that promoter DNA hypermethylation dependent transcriptional repression of specific genes, *viz*., *GSTU10*, *MAPK12*, and *BXL1*, primarily mediated by *METHYLTRANSFERASE1* (*MET1*) and *DOMAINS REARRANGED METHYLTRANSFERASE2* (*DRM2*) methyl transferases, is an essential criteria for the establishment of undifferentiated state in callus cells (Figure 6) (Berdasco et al., 2008). There are also studies which show the association of promoter DNA hypomethylation mediated upregulation of several members of the NAC (NAM/ATAF1/*CUC2*) domain family with the acquisition of pluripotency in the protoplast cells of leaves (Avivi et al., 2004). This implies that the establishment and maintenance of undifferentiated state in plant cells necessitates both upregulation and downregulation of genes brought about by changes in the levels of DNA methylation. PICKLE (PKL) is a pivotal chromatin remodeler which belongs to Chromodomain-Helicase-DNA binding3 (CHD3)/CHD4 family of proteins (Eshed et al., 1999; Ogas et al., 1999). Recent studies elucidated the role of PKL as a negative regulator of cytokinin response during regeneration as the *pkl/cytokinin hypersensitive2* (*ckh2*) mutant displayed sensitivity to low levels of cytokinin and proliferated into green colored calli at considerably lower levels of cytokinin, compared to wild type (Furuta et al., 2011).

During *de novo* shoot regeneration, *WUS* plays a key role as its domain of expression specifies shoot organizing center, which confers stemness to the neighboring cells (Schoof et al., 2000; Gallois et al., 2004; Gordon et al., 2007). Thereby, *WUS* expression can be positively correlated to *de novo* shoot regeneration. Recent studies conducted by Li et al. (2011) illustrated the striking correlation between the epigenetic control on levels of *WUS* expression and developmental rates of *de novo* shoot regeneration. For this, they exploited mutants defective in the following salient epigenetic regulators involved in DNA methylation and histone modifications viz., *MET1*, encoding for DNA methyltransferase, *KRYPTONITE* (*KYP*) for the H3K9 methyltransferase, *JMJ14* for H3K4 demethylase, and *HAC1* for the histone acetyl transferase. The *met1*, *kyp*, and *jmj14* mutants are associated with epigenetic changes involving increased transcriptional activation of their downstream targets and they displayed precocious regeneration of *de novo* shoots. Meanwhile *hac1* mutant, associated with more repressed transcription showed delayed developmental rate of *de novo* shoots when the individual mutant calli were placed on SIM. Strikingly, the levels of *WUS* expression was significantly enhanced in *met1*, *kyp*, and *jmj14* mutant calli while it was reduced in *hac1* mutant calli when compared to wild type calli at same time points on SIM, suggesting that these epigenetic regulators control the developmental rates of *de novo* shoot regeneration by dynamically regulating *WUS* expression (Figure 6) (Li et al., 2011).

As already described, somatic embryogenesis is a regenerative process; wherein the differentiated somatic cells dedifferentiate to form somatic embryos that further develop into complete plantlets. *clf swn* double mutant exhibits somatic embryo formation from masses of undifferentiated cells formed on the plantlet tissues (Chanvivattana et al., 2004). This is a spectacular example for the involvement of PRC2 components in the maintenance of differentiated state, which is in contrast to their role in the acquisition of pluripotency as stated earlier. *PKL* is also implicated in the maintenance of differentiated state in plants and acts via suppression of *LEC1*, an activator of embryonic development (Ogas et al., 1999). *pkl* mutant exhibits characteristics of embryonic tissue in the primary root meristem, which would grow thick and become opaque and green after an initial period of normal growth and the primary roots are named as pickle roots. The *pkl* roots, when excised and cultured on a normal MS medium without the exogenous supply of plant hormones, showed development of somatic embryos (Ogas et al., 1997). These findings entail the potency of *PRC2* components and *PKL* to enable the cells sustain the state of differentiation as their loss of function evoke cellular reprogramming resulting in the acquisition of a dedifferentiated state, leading to the development of somatic embryos.

These leading bodies of experiments performed in *Arabidopsis*, gives a flavor of the diversifying and also contrasting roles of chromatin remodeling during regeneration. These involve the maintenance of differentiated state, the loss of differentiated state and acquisition of pluripotency and dynamic regulation of gene expression during *de novo* shoot formation. However, the complete picture on the intricate entanglement of the players of chromatin remodeling with each step of regeneration in plants is yet to be unraveled.

## Regeneration: an evolutionary perspective

Regeneration is a process that is spread in the domain of life and the strategies employed for regeneration show a striking similarity across kingdoms (Figure 1). When the specialization of various cells to carry out different functions required for a multicellular organism gives a convincing reason for the confinement of regenerative cells to their niches, it is quite surprising to see that the ability to divide is compartmentalized even in prokaryotes. Bacteria were long thought to be following identical division producing daughter cells that are competent for further divisions. But *Caulobacter crescentus*, an α-proteobacterium divides asymmetrically producing a sessile stalk cell and a motile swarmer cell. The replication of the motile cell is under tight regulation of *CtrA*, which represses cell division by binding to origin of replication (Domian et al., 1997; Quon et al., 1998). Once proper signals are received, *CtrA* is dephosphorylated and degraded thereby mediating the cell fate change to the stalk cell which is competent for reproduction (Domian et al., 1997; Bastedo and Marczynski, 2009). As described earlier, epigenetic control which play a crucial role in determining the competence for regeneration in plants is active even in bacteria (Domian et al., 1997). The reprogramming of the swarmer cell to stalk cell can be seen as the primordial form of reprogramming that is seen during tissue regeneration in higher plants and animals. And the asymmetric division seen in the bacteria like *Caulobacter* is one of the most primitive modes of reproductive asymmetry that is seen in higher plants and animals, where a specialized group of cells (stem cells) retain their ability to divide perpetually without getting committed to a specific fate while other loses competence to divide indefinitely and differentiates into specific fates. All these evidences point us to the conclusion that strong evolutionary forces are responsible for controlling the regeneration of an organism. The spatio-temporal localization of the stem cells which plays an important role in determining the regenerative biology of an organism is modulated in different organism through the course of evolution.

Algae are the simplest members of the plant kingdom. The taxa contain diversity of species that may either be single cellular or multicellular showing striking ability of regeneration. Most of the multicellular algae just consist of a structure called thallus which is a mass of undifferentiated cells, but have structures analogous to plant organs of higher plants (Goldstein, 1973). When most of the other multicelluar algae are competent to repair wounds by regenerating new cells and even regenerate an entire algal body from explants containing just a few cells under natural conditions (Aguirre-Lipperheide et al., 1995; Huang and Fujita, 1997) a few members of the division, like *Acetabularia* goes a step ahead by regenerating the wounded cell (Mandoli, 1998). The animal counterpart for algae would be phylum porifera, which exhibits a simple body plan with very few cell types. As in algae the sponges are also able to regenerate upon wounding or an entire organism from just a lump of cells (Hoppe, 1988; Duckworth et al., 2003).

Interestingly, bryophyte which exhibits more complex body plan than the algae follows a similar pattern in regeneration. The body plan of the haploid embryophyte of bryophytes which occupies the major phase of its life cycle also consists of thallus. So the number of lineages of cells is highly limited. This could be the reason for the remarkable regenerative ability of mosses, where they can regenerate any wounded tissue or an entire plant from the powdered explants without any external hormone supply (La Farge et al., 2013). The mode of regeneration exhibited by cnidarians (includes jellyfish, sea anemones, and corals) is strikingly similar to that exhibited by bryophytes (Lenhoff and Lenhoff, 1986). They have a simple body plan like algae with just two germ layers and a few cell lineages. As in the algae, the cnidarians can also regenerate an entire organism from just a clump of cells (Noda, 1971; Gierer et al., 1972; King and Newmark, 2012). Even dissociated single cells can rejoin to regenerate an entire organism. Seemingly the competence for regeneration is spread throughout body of these organisms with relatively simpler cellular organization (Gierer et al., 1972; King and Newmark, 2012).

In pteridophytes, the sporophytic generation which occupied a very small part of the life cycle of algae and bryophytes has equal representation as the gametophyte. Thus, the regenerative competence of sporophytes was put to test in these taxa of plants. Intriguingly in ferns, plant explants of the gametophyte showed remarkable potential for regeneration in hormone free medium (Banks, 1999; Kazmierczak, 2003; Menendez et al., 2009; Abul et al., 2010; Somer et al., 2010). While in the sporophyte though the potential for regenerating an excised organ remained, the explants were recalcitrant to regenerate an entire organism when no external hormone is supplied (Ferna'ndez et al., 1993, 1997). The callus induction by hormones was required for reprogramming the cells to a competent state.

The sporophyte of flowering plants is the most complex division in the plant kingdom. Unlike all other divisions, they form many organs and have many lineages of cells specialized for various purposes. This specialization comes with a price on the regenerative potential of the plant. In angiosperms, not many cell lineages are competent for regeneration and the ones that can form an entire plant from explants in a hormone free medium are highly confined to few niches (Atta et al., 2009). But even in angiosperms the highly diminished gametophytes retain their regenerative potential as evident from pollen grain which can undergo embryogenesis under stressful without external hormones (Reynolds, 1997; Segui-Simarro et al., 2011; Soriano et al., 2013). Dicotyledonous plants like *Arabidopsis* have cambium stem cells localized to specific niches in their body plan that helps in secondary thickening of the plant. But monocots that evolved relatively recently from dicots lack the cambium meristem and have a limited amount of meristematic cells in their body plan thereby narrowing the regenerative potential. The story in animals is also not very different. In humans, embryonic stem cells retain the ability to regenerate entire organisms while all other cells have lost their competence to regenerate a complete individual (Wong et al., 2011) But some residual competence is retained by stem cells present in the adult body which does the maintenance and repair of the bodily organs as it is done in plants.

As the process of evolution produces more and more complex organisms with complex organs and organ systems, myriads of cell lineages dedicated for specific processes, the reproductive potential is traded off. The competence for regeneration is tightly controlled in higher organisms and external agents are required to reinstate regenerative potentials to a differentiated cell. This is the common feature seen in both plants and animals. Further, localizations of regenerative cells are restricted spatially and temporally during the organismal development in both animals and plants.

## Future perspectives

Elegant set of experiments using QC ablation in the root meristem and regeneration from cut meristem shed light on contribution of stem cells maintenance in regeneration (Xu et al., 2006; Sena et al., 2009). However, absolute necessity of stem cells for regeneration yet needs to be demonstrated, in particular after decapitating the root meristem. It is tempting to speculate that stem cells are required for regeneration and partial maintenance of stem cells can still lead to regeneration as it was seen in the experiments done by Sena et al. (2009). Intriguingly, only sub sets of root meristem cells located in vicinity of stem cell niche have potential to regenerate rest of the meristem after decapitation suggesting sheer ability to divide may not suffice to provide regeneration competence. Combination of cell fate determining factors, epigenetic regulators, and dynamic accumulation as well as synthesis of plant hormone are likely to be the key players. Careful in depth analyses of differences in the molecular environment between the root meristem cells with ability to regenerate and cells which lack regeneration potential will be instrumental to understand the molecular mechanisms to establish the competence.

Remarkable ability to regenerate entire organism from different explants has been exploited for micropropagation of various plant species. However, unlike animal kingdom, molecular insights into plant regeneration remained largely unknown till recently. Present studies have begun to uncover molecular nature of competence and order of events during regeneration in model dicot species Arabidopsis thaliana. One of the most recent striking finding is the nature of callus which was long thought as dedifferentiated tissue. Expression patterns of genes combined with genetic studies suggest that callus is root like tissue and callus originates from pericycle like cells from various explants including some of the aerial organs such as cotyledons and petals (Sugimoto et al., 2010). It will be revealing to include explants like stem and leaves of different plant species to evaluate whether cells other than pericycle like cells also participate to generate callus mass and whether these other cells also pass through competence state resembling pericycle-like cells.

It is important to note that callus is not only derived upon external hormone application but it can also be formed upon wounding (Pang et al., 2008; Zhang et al., 2011). An interesting question is whether callus derived upon wounding have potential to regenerate entire plant body plan. Recent studies demonstrate that *Arabidopsis* WIND1, which is involved in wound-induced callus (Iwase et al., 2011), can also trigger callus formation in other plants species. An important step ahead would be to utilize these calli as tools to probe their competence to regenerate complete plant body. Despite the fact that we have begun to understand the callus identity, how cells of initial explants acquire competence to generate pluripotent callus, remains elusive. Furthermore, it will be crucial to unravel how the competent callus is generated and how various regulatory interactions necessary for shoot formation are assembled on rough callus surface.

### Conflict of interest statement

The authors declare that the research was conducted in the absence of any commercial or financial relationships that could be construed as a potential conflict of interest.
